# Epistemic Consequentialism, Truth Fairies and Worse Fairies

**DOI:** 10.1007/s11406-017-9833-0

**Published:** 2017-04-12

**Authors:** James Andow

**Affiliations:** Department of Philosophy, University of Reading, Reading, RG6 6AA UK

**Keywords:** Epistemic consequentialism, Rationality, Truth fairy, Worse fairy

## Abstract

Direct Epistemic Consequentialism faces the Truth Fairy. Indirect Epistemic Consequentialism promises to avoid this issue. But there are worse fairies than the Truth Fairy. There is the Worse Fairy. The case of the Worse Fairy helps demonstrate that epistemic consequentialists who would solve problems like the Truth Fairy by ‘going indirect’ face a dilemma.

## Epistemic Consequentialism

Consequentialist theories in ethics combine an axiology and a criterion which tells us what is morally right (or permissible). What’s valuable? Perhaps, utility. Perhaps, something else. What’s permissible? Perhaps, only actions which maximize utility. Perhaps, something else. What makes a moral position consequentialist is that it holds that the correct criterion ultimately grounds moral rightness and wrongness of actions in the consequences of those actions.

Consequentialist theories in epistemology are best understood as doing something similar. They combine an axiology and a criterion which tells us what is epistemically right (or permissible). What’s valuable? Perhaps ultimately, true beliefs. But, perhaps, justification too. Perhaps, knowledge. Perhaps, something else. What’s permissible? Perhaps, only forming beliefs which will (compared with the alternatives) make your situation epistemically better. What makes an epistemic position consequentialist is holding that the correct criterion ultimately grounds epistemic permissibility of our epistemic goings on, e.g., our adoption of beliefs, in the consequences of those goings on. As Ahlstrom-Vij and Dunn (2014) put it, ‘the epistemically right (e.g., the justified) is to be understood in terms of conduciveness to the epistemic good (e.g., true belief)’.

Those who have explored and discussed consequentialist approaches in epistemology have typically framed things solely in terms of the epistemic status of beliefs or the acceptance of propositions (see, e.g., Briesen 2016; Percival 2002). Epistemic consequentialism has been characterized as the view that ‘epistemic features such as warrant accrue to a belief (or acceptance) of S’s in virtue of the (expected) epistemic consequences of S’s being in that state’ (Elstein and Jenkins 2017) or the view that ‘the epistemic status of an attitude is determined by the epistemic value of its consequences compared to the epistemic value of the consequences of the alternatives’ (Jenkins 2007). But there is no obvious reason to keep things so restricted. These are not the only activities which are plausibly governed by epistemic norms.

Keeping things more open, we can characterize a simple direct form of epistemic consequentialism as endorsing the following criterion of epistemic permissibility.

**DEC** X is epistemically permissible iff X maximizes expected epistemic value^1^

This is, of course, not the only criterion the consequentialist might endorse. For example, they might be a satisficing epistemic consequentialist. However, here, I’ll restrict my discussion to those who endorse a maximising form of consequentialism.

The remainder of the paper proceeds as follows. §2 introduces a particular type of problem which direct epistemic consequentialism faces and explains how indirect consequentialism promises to provide a solution. §3 then describes a novel, similar variety of problem using the Worse Fairy case which faces the indirect consequentialist. Finally, §4 draws out a number of lessons. In particular, I will argue that what cases such as the Worse Fairy make clear is that the epistemic consequentialist who tries to avoid the problems described in §2 by ‘going indirect’ faces a dilemma. I’ll take this to suggest that that indirect consequentialism is not an appropriate solution to the intuitive problems such as those highlighted by the Truth Fairy case that epistemic consequentialism faces.

## Truth Fairy and Indirect Consequentialism

Direct versions of epistemic consequentialism (which endorse something like DEC) face certain counterintuitive implications. This problem can be highlighted using cases such as the Truth Fairy (based on Elstein and Jenkins 2017).

**Truth Fairy** Suppose you start with no reason to believe that p is true and no reason to believe that it is false. The Truth Fairy is a very powerful being, and she makes you the following credible offer: you accept p as true, and she will make your epistemic situation very, very good overall. She will arrange for you to have many, many true, justified, knowledgeable beliefs, and very, very few false, unjustified or unknowledgeable ones. However, she does not guarantee that your trust in p itself will have any particular epistemic status as a result of her actions. By DEC, her offer makes trust in p permissible. But intuitions rebel at this thought.

The problem is that according to DEC it is *epistemically* permissible to accept that p, which is really rather counterintuitive. The Truth Fairy is not alone. It is but one instance of a more general pattern. Intuitions are pretty clear on the idea that epistemic permissibility is not simply a matter of which activities will improve one’s overall epistemic position.

How do you solve a problem like the Truth Fairy? One proposed solution takes a familiar pattern. When direct forms of consequentialism face the problem of counterintuitive implications, very often indirect forms look like a promising way to deal with such problems without giving up on consequentialism. The same is true in the case of epistemic consequentialism and cases such as the Truth Fairy’s (pace concerns raised by Elstein and Jenkins which I’ll discuss in a moment). An indirect form of epistemic consequentialism seems to be able to avoid the implication that trusting in p is epistemically permissible in the Truth Fairy case.

To see the promise in this solution, consider the following criterion which an indirect epistemic consequentialist might endorse (in this case the indirect consequentialism takes the form of a rule consequentialism):

**REC** X is epistemically permissible iff X is allowed by the rule set R the internalization of which maximizes expected epistemic value

REC might seem to be able to avoid the relevant implications. Following any rule which would permit, for example, adopting beliefs on the basis of offers like the Truth Fairy’s is intuitively not a good way to go about adopting beliefs. It seems that no such rule can be epistemically optimal. This ‘going indirect’ solution has been defended notably by Elstein and Jenkins (2017).

Elstein and Jenkins address a further worry which one might have about whether ‘going indirect’ can provide an adequate solution. This additional worry is that, even though indirect epistemic consequentialism can avoid counterintuitive implications such as those highlighted in the Truth Fairy case, it can’t give a satisfactory explanation as to *why* forming such beliefs is epistemically impermissible. They consider the worry that:

1. REC says that taking up offers like the Truth Fairy’s is a bad way to form beliefs *only for individuals who happen to be in particular circumstances*, for example, in which there are no Truth Fairies.^2^ In worlds in which Truth Fairies abound, surely accepting offers like the Truth Fairy’s would maximise epistemic utility.
2. But, taking up offers like the Truth Fairy’s is intuitively not epistemically respectable *regardless of contingent facts about the preponderance of Truth Fairies*.

This additional worry is a general worry for indirect varieties of epistemic consequentialism (not just a worry for indirect consequentialism qua solution to problems like the Truth Fairy). Any rules which allow doing X if it will maximise your overall position even if X itself has no positive epistemic value are rules which will be beneficial rules to follow in certain worlds and not in others. The worlds in which adopting such rules is beneficial will be worlds in which apparent opportunities to maximise your epistemic position by doing such things tend to be more than merely apparent. At heart, indirect consequentialism doesn’t do justice to our intuitions about such things since doing such things is intuitively not epistemically respectable *regardless of contingent facts about the precise structure of the epistemic environment*.

Elstein and Jenkins attempt to address this worry in the specific case of the Truth Fairy. They argue that 1 is false. They claim that *even were Truth Fairies to abound* it would not maximise expected utility ‘to (attempt to) internalize a rule that recommends accepting propositions without evidence in response to Truth-Fairy-like offers’. Their argument relies on factoring in (what they take to be) the extremely high ‘internalization costs’ involved. They think that, given our current practices and internalized epistemic rules, we simply can’t accept the Truth Fairy’s bargain. They think that we can’t accept a belief we take ourselves to have no (fairy independent) reason to accept. They think that the costs involved in changing those practices and internalizing different rules would be so high as to mean that to maximize expected utility one should not attempt to take the Truth Fairy’s bargain and so taking the bargain is not epistemically permissible.^3^ And similar considerations will apply for any epistemic rule which permits doing X if it will maximise your overall position even if X itself has no positive epistemic value.

## The Worse Fairy

Whether or not indirect epistemic consequentialism has the resources to avoid the type of counterintuitive implications highlighted by the Truth Fairy case and to address the further worry considered by Elstein and Jenkins, I argue there is a more important worry lurking. There are worse fairies than the Truth Fairy. Indeed, there is the Worse Fairy.

**Worse Fairy** Suppose you start with no reason for internalizing a rule set, R1, governing belief adoption. The Worse Fairy is a very powerful being, and she makes you the following credible offer: you internalize R1, and she will make your epistemic situation very, very good overall. She will arrange for you to have many, many true, justified, knowledgeable beliefs, and very, very few false, unjustified or unknowledgeable ones. There is no guarantee and no particular reason to think that internalizing R1 would, without the Worse Fairy’s bargain in play, have any particular positive consequences.

Why is the Worse Fairy so nasty? Why is the Worse Fairy worse? The reason is that the case helps to draw out some counterintuitive implications of indirect forms of epistemic consequentialism. First, consider that any rule which is a plausible candidate for a rule to appear on the right hand side of REC, must allow its own adoption (otherwise it wouldn’t be permissible to adopt it). Second, consider the rule set, R1, which the Worse Fairy suggests you adopt. What are the consequences of internalizing R1? The consequences are that the Worse Fairy will make your epistemic situation very, very good overall, viz., epistemic value will be maximized. Thus, by REC, internalization of R1 on the basis of the fairy’s offer is epistemically permissible. But, as in the case of the Truth Fairy and DEC, I take it this consequence is very counterintuitive.

In essence, the problem highlighted by the Worse Fairy case is a general one facing indirect rule epistemic consequentialists: accepting a rule simply because doing so is the way to maximise your overall epistemic position even when there is no reason to think that following the rule will in itself improve your epistemic position in anyway is intuitively problematic. One way to bring out the degree to which this is counterintuitive is to consider some rules which the Worse Fairy could ask you to adopt: apportion your trust in a source according to the colour of their shoes; form no beliefs about your own personality on a Tuesday; allow whether a proposition can be expressed in iambic pentameter to modify the credence one assigns to it, and so on. Adopting these rules is clearly epistemically problematic even if, in Worse Fairy conditions, doing so would result in maximising one’s overall epistemic position. The fact that adopting a rule will maximise one’s overall epistemic position clearly gives you *some* kind of reason to internalize the rule. However, it simply doesn’t make it that this is an *epistemically* laudable or permissible thing to do. In fact, in circumstances such as those involving Worse Fairies, it looks epistemically irresponsible (even if it is to be recommended all things considered).

## A Dilemma Looms

What lessons should we take from the Worse Fairy and problems like her?

The first lesson is that although a proposed way to avoid counterintuitive implications at the level of the adoption of beliefs, e.g., to avoid the Truth Fairy, might be successful, it might fail to provide the resources to solve what is essentially the same problem at the level of the adoption of rules, e.g., to avoid the Worse Fairy. Elstein and Jenkins’s sophisticated defence of REC’s ability to say sensible things about the Truth Fairy is an example which makes this point clear. Their defence of REC in the face of the Truth Fairy cannot be extended in order to defend against the Worse Fairy. Elstein and Jenkins’s defence doesn’t provide resources which would allow them to say that it is epistemically impermissible to adopt rules simply to maximize one’s epistemic position. Their defence leans on the issue of high internalization costs. The internalization costs associated with internalized new rules and practices *that would allow one to accept a proposition you take yourself to have no reason to believe* may be pretty high (let’s grant them this and that thus their defence of REC’s ability to avoid the Truth Fairy goes through). However, there is no obvious reason to think the costs associated with *internalizing a rule without taking ourselves to have a fairy-independent reason for doing so* are similarly high. Indeed, to my mind, the internalization costs in the latter case would be considerably lower. Internalizing such rules isn’t generally associated with any great *epistemic* cost.^4^

The second lesson is that this means we can place a condition on the success of proposed solutions to the Truth Fairy problem and problems like it. Unless one’s proposed solution to avoid counterintuitive implications at the level of the adoption of beliefs, e.g., to avoid the Truth Fairy, also provides the resources for solving analogous problems at the level of rules, such as the Worse Fairy, then it is not really even an adequate solution to the first type of problem.

You might question this. And, of course, it is in some sense *open* to the indirect epistemic consequentialist to use one set of resources to solve problems like the Truth Fairy and come up with an independent response to problems like the Worse Fairy. But that kind of approach looks objectionably ad hoc. The two types of problem are really two sides of the same coin. The two problems both feed off same basic intuition that X can’t be *epistemically* permissible simply in virtue of the fact that the consequences of doing X will put you in the best epistemic position, whether X be accepting a belief or adopting a rule governing belief formation. If the epistemic consequentialist aims to avoid problems like the Truth Fairy by going the indirect route, they need to do so in a way which also avoids problems like the Worse Fairy.

There is, of course, the bullet biting option. This option involves not buying into the relevant intuition that adopting a rule in a case like that of the Worse Fairy would be impermissible (or, at least, finding some way to legitimately get away without accommodating it). For example, one could give up on the intuition that adopting rules in Fairy ways is not epistemically respectable.^5^ However, this move seems suspect. Since it makes the move to indirect consequentialism look superfluous. If one is willing to bite bullets at the level of problems like the Worse Fairy then there is no obvious reason why one shouldn’t have been willing to bite bullets at the level of problems like the Truth Fairy in the first place—an option discussed by Elstein and Jenkins —and, of course, if you had done that there would be no need for ‘going indirect’.

So, the third and final lesson is that we have something like a dilemma looming for the indirect consequentialist which is drawn out by cases like the Worse Fairy. On the first horn, the indirect consequentialist tries to solve problems like the Worse Fairy with different resources than they use to solve problems like the Truth Fairy (because they go indirect to solve issues like those highlighted by the Turth Fairy case but going indirect doesn’t by itself solve issues like those highlighted by the Worse Fairy case). The unacceptability of this horn is that the solution then looks ad hoc; since both types of the problem are the same at heart, they really ought to be solved in the same way. On the second horn, the indirect consequentialist tries to avoid the Worse Fairy types of issue by biting the bullet or otherwise excusing themselves of accommodating the relevant intuitions. The unacceptability of this horn is that bullet biting would also have solved the problems which motivated the move to indirect consequentialism and, since the basic intuitions underlying problems such as the Truth Fairy and the Worse Fairy are the same, there is no obvious motivation for taking different strategies vis-á-vis bullet-biting to the two types of problem. This dilemma suggest to me that indirect consequentialism is not an appropriate solution to the intuitive problems, like the Truth Fairy, that epistemic consequentialism faces.^6^

## Footnotes

1 The wording of DEC, the Truth Fairy case, and REC is based on that in Elstein and Jenkins (2017). In each I avoid talk of ‘warrant’ as this is doesn’t natually extend to other epistemic goings on other than belief, etc. Instead, I use ‘epistemically permissible’ to capture the same idea but in a way that more naturally extends to other epistemic goings on.

2 Or, I suppose, in which there are some but they are vastly outnumbered by things resembling Truth Fairies but are not Truth Fairies

3 Now, in fact, although it isn’t essential to my point, I don’t think Elstein and Jenkins ’s argument works. Note that the relevant internalization costs which sway the balance must be *epistemic costs*. But, will there be fewer true beliefs, justified beliefs, less knowledge? I can’t see how – given that we can beef up the Truth Fairy’s offer to make one’s epistemic situation infinitely good, or at least as good as one’s poor little head can possibly take.

4 This is especially clear given their own minimal understanding of what counts as having internalized a rule: “Any practice which is second nature, but which is still evaluable in terms of reasons, has to be understood as rule-driven, even though, because the rules are internalized, the application of the rules is merely implicit” (Elstein and Jenkins 2017).

5 One way to do this might be in reliabilist terms (see Ahlstrom-Vij and Dunn 2014’s attempt to defend a reliabilist response to cases from Berker 2013 which are somewhat similar to the Truth Fairy case). Reliabilists have options concerning with respect to which possible worlds the reliability of our epistemic goings on get evaluated (see Goldman and Beddor 2016 for an overview). But, one option available to them is to say that it is reliability in our world, sans Fairies—or in normal worlds which are relevantly similar to ours, e.g., no Fairies—which matters to the evaluation of our epistemic goings on.

6 Of course, this in no way impugns other proposed solutions to the Truth Fairy problem and problems like it which do not rely on indirect consequentialism. It is worth noting that I have only discussed rule versions of indirect consequentialism in this paper. But hopefully it should be clear that other types of indirect consequentialism will face analogous cases to that of the Worse Fairy and thus face analogous dilemmas.
